# Paying Patients at Cardiff Infirmary

**Published:** 1911-12-30

**Authors:** Leonard D. Rea

**Affiliations:** Secretary and General Superintendent, King Edward VII.'s Hospital, Cardiff.


### PAYING PATIENTS AT CARDIFF INFIRMARY.

### How the New System was Instituted.

By LEONARD D. REA, Secretary and General Superintendent, King Edward VII.'s Hospital, Cardiff.

Of the many suggestions advanced by Colonel Bruce Vaughan, the Chairman of the House Committee, with a view to increasing the income of the Cardiff Infirmary, probably none was more unpopular than the recommendation that each in-patient should be asked to pay a small sum towards the cost of his food (or maintenance), and each out-patient be charged a nominal fee for registration. Eventually, in February last, the Board of Management decided to adopt the principle as an experiment for twelve months, and on Monday, April 3, the system was quietly and unobtrusively put in operation. The writer would desire to express his great indebtedness to the Secretaries of the London and Guy's Hospitals for the valuable information so generously afforded by them regarding the systems in practice at their Institutions, and which have been closely followed in respect to the charges made to out-patients.

### How the Patients' Support was Won.

For some weeks previous to the enforcement of the scheme, notices were placed in the wards and out-patient department informing patients of the proposed charges, and the patients and their friends were also advised by verbal communications of a more explanatory and less official character. An intimation of the proposed charges was also added to the usual notice advising a patient of the date when he would be admitted. A few days before the scheme came into operation, I made little speeches in each ward, explaining the matter and inviting questions if any misunderstanding existed, and I am gratified to say that in this manner I won the whole-hearted co-operation of those patients who were well enough to appreciate what I said. It may be of interest to give the substance of my remarks, as indicating the aspects of the question which proved acceptable to the patients.

First of all, I made it clear that we asked them to pay only a small proportion of their actual cost to the Institution, namely, 6d. towards the 3s. 6d. per day which represents the average cost of each in-patient's "maintenance." I deprecated the term "food," which had been printed in the earlier notices, as an impression had been created that by reason of the payments the quality of the food might be altered, or a patient permitted a more varied choice. I substituted the term "maintenance," pointing out that "food" had originally been employed to make it quite clear that the charge is in no respect made in regard to medical service, which is, of course, given without money and without price. I intimated that no charge would be made to those patients who really could not afford to pay, and that the Sisters of the wards would ascertain such inability but respect the confidence, so that no distinction should be made between those who paid and those who did not. Finally, I appealed for their sympathy and co-operation for sufferers, just like themselves, and of whom no fewer than 700 were at that time waiting for admission to the Hospital.

The machinery for collecting the in-patients' payments is simple and owes its success to the kindness of the Sisters of the wards, who have voluntarily undertaken their collection. Payments are made in advance for a whole week—from Monday to Sunday—on each Monday morning, and the Sister gives for each 3s. 6d. a printed receipt, consecutively numbered, on which the following notice is also printed: "In the event of a patient being discharged before the Sunday, the Secretary will, if requested, return to the patient an amount equivalent to the payments for unexpired days." Patients have rarely availed themselves of this provision, as they are generally pleased to leave the little balances as tokens of their gratitude. Receipts on a different coloured paper are given in respect of the broken parts of a week, when patients are admitted during the period between Monday and Monday, but all payments are made in advance, either for the week or a lesser period. The Sisters have had little difficulty in ascertaining whether a patient is able to pay, but refer any doubtful cases for my consideration. We treat with the utmost tenderness those who really cannot pay, but are quite firm when we have reason to believe a patient is able to pay but is reluctant to do so. Occasionally a patient expresses a wish to pay a little more, perhaps 5s. a week.

### The Out-Patients' Charges.

The system of charging out-patients has worked quite as smoothly as that relating to in-patients. The official who receives the payments is entrusted with books of consecutively numbered adhesive stamps, each stamp bearing a face value of 3d., and the patient, upon applying at the Out-patient Registration Office, is given one of these stamps in exchange for 3d. This is affixed in his presence to his prescription card. The dispenser has instructions, before dispensing medicine, to see that the prescription card bears the adhesive stamp and the date of attendance, or that it is impressed by the official rubber stamp, "Free," used when patients cannot afford the registration fee.

It is a matter for congratulation and the answer of actual experience to the criticisms which were advanced against Colonel Bruce Vaughan's advocacy of this system that, for the eight months ended November 30 last, the receipts from this additional source of income have amounted to £823 11s.—£494 0s. 6d. from in-patients and £329 10s. 6d. from out-patients. What perhaps may prove of greater importance than the immediate financial advantage is the fact that the system has so far been adopted without apparent vexation or hardship, and in many instances with the expressed approval and good-will of the patients themselves.

